# Mantle Subduction and Uplift of Intracontinental Mountains: A Case Study from the Chinese Tianshan Mountains within Eurasia

**DOI:** 10.1038/srep28831

**Published:** 2016-06-29

**Authors:** Jinyi Li, Jin Zhang, Xixi Zhao, Mei Jiang, Yaping Li, Zhixin Zhu, Qianwen Feng, Lijia Wang, Guihua Sun, Jianfeng Liu, Tiannan Yang

**Affiliations:** 1 Institute of Geology, Chinese Academy of Geological Sciences, Beijing 100037, China; 2 School of Ocean and Earth Sciences, State Key Lab of Marine Geology, Tongji University, Shanghai 200092, China; 3 Department of Earth and Planetary Sciences, University of California, Santa Cruz, California 95064, USA

## Abstract

The driving mechanism that is responsible for the uplift of intracontinental mountains has puzzled geologists for decades. This study addresses this issue by using receiver function images across the Chinese Tianshan Mountains and available data from both deep seismic profiles and surface structural deformation. The near-surface structural deformation shows that the Tianshan crust experienced strong shortening during the Cenozoic. The receiver function image across the Tianshan Mountains reveals that the lithosphere of the Junggar Basin to the north became uncoupled along the Moho, and the mantle below the Moho subducted southwards beneath the northern part of the Tianshan Mountains, thereby thickening the overlying crust. Similar deep structures, however, are not observed under the Tarim Basin and the adjacent southern Tianshan Mountains. This difference in the deep structures correlates with geomorphological features in the region. Thus, a new model of mantle subduction, herein termed M-type subduction, is proposed for the mountain-building processes in intracontinental compressional settings. The available geomorphological, geological and seismic data in the literatures show that this model is probably suitable for other high, linear mountains within the continent.

Mountains and basins are two types of basic tectono-geomorphologic units on the Earth. How and why mountains on continents uplift has challenged geologists since the late 1800s^1^. With the tremendous advance of the theory of the plate tectonics, geologists now recognize that mountains on continental margins, such as those around the Pacific ocean, are products of the subduction of oceanic lithospheric plates under continental plates (B-type subduction, for short), and that intercontinental mountains, such as the Himalayas and Alps, are the products of the subduction of one continental plate under another (A-type subduction). However, the origins of some intracontinental mountains, such as the Petermann and Alice Springs orogens in central Australia and the Tianshan Mountains in central Eurasia, still puzzle geologists^2^,^3^,^4^,^5^,^6^,^7^,^8^. Does the uplift of intracontinental mountains originate from the subduction of lithospheric plates similar to beneath continent-marginal and intercontinental mountains? If so, what type of subduction occurs? Does the entire lithospheric plate subduct, similar to A-type subduction between two continents, or does only the mantle part of the lithosphere subduct?

This study focuses on the Chinese Tianshan Mountains (Segment C in Fig. 1B) to discuss the driving mechanism that is responsible for the uplift of linear intracontinental mountains by using data on deep structures as revealed by seismic receiver function images in combination with geomorphologic features, structural deformation near the surface, and other available seismic data.

## Geography and Regional Geology of the Tianshan Mountains

**Figure 1:**
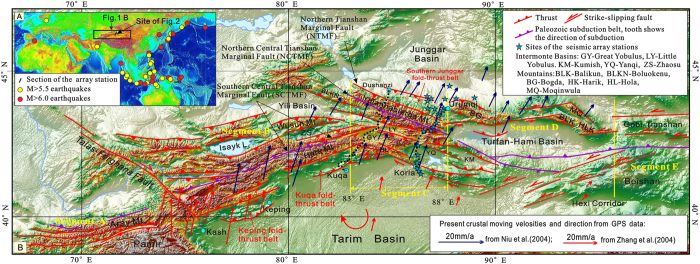
Geomorphological and Tectonic features of the Tianshan Mountains. (**A**) Study area and earthquakes that were used for the formation of the receiver function image, which were selected from more than 500 earthquakes from a USGS database that was created during this study’s data collection. (**B**) Geomorphologic and tectonic features of the Tianshan Mountains, which show their segmentation with latitude and zoning with longitude, Cenozoic faults^36^ and Paleozoic subduction zones^9^,^11^,^12^,^13^,^14^,^62^, the asymmetry of structural deformation near the surface on both sides^12^, the crust’s velocity and direction from GPS data^60^,^61^, and the clockwise rotation of the Tarim Blocks^22^,^40^. The primary DEM data that were used for the geomorphological features in (**B**) are in the SRTM GTOPO 30 format and were provided by NASA and downloaded from http://glcf.umiacs.umd.edu in 2010. The figure was generated using ArcMap v10.1 (http://www.esrichina.com.cn/softwareproduct/ArcGIS/) and processed by CorelDRAW X7 (http://www.coreldraw.com/cn/product/graphic-design-software/?hptrack=cn2hr1&_ga=1.59748394.665041398.1450858064).

The Tianshan Mountains in the interior of the Eurasian continent extend from Uzbeckstan eastwards through Kyrgyzstan and Northwest China to southwestern Mongolia, stretching nearly 3000 km in length and 200–400 km in width. This range exhibits distinctive geomorphological characteristics, and can be subdivided into five segments from east to west: Segment A, which is located west of the NW-trending Talas-Ferghana fault, features a southward arc-like linear mountain range; Segment B features NE-trending linear mountains to the south and alternating ranges and basins to the north; Segment C exhibits the opposite geomorphologic framework as Segment B; Segment D features a high, curved mountain range to the north, a single large basin in the center, and lower hills and the Gobi Desert to the south; and Segment E features lower hills and desert basins (Fig. 1B). Segments C and D, and most parts of Segments B and E in the mountains are located within China, between the Junggar Basin to the north and the Tarim Basin to the south. These features possibly imply a variety of origins and uplift mechanisms for these intracontinental mountains.

Geologically, the crust of the Tianshan Mountains is composed of a mosaic of Paleozoic oceanic and island arc fragments^9^,^10^,^11^,^12^,^13^,^14^. Mesozoic and Cenozoic terrestrial sediments accumulated in intermontane basins in the Chinese part of the mountains, and Cenozoic thrust faults are present along the boundaries of all the basins^15^,^16^,^17^,^18^,^19^,^20^,^21^ (Fig. 1B). Interestingly, a Cenozoic basin-verging fold-and-thrust belt that is similar to that in the foreland basin of the collisional orogenic belt occurs in the piedmont of the linear mountains in Segments B and C^22^,^23^,^24^,^25^,^26^,^27^,^28^,^29^,^30^,^31^,^32^ (Fig. 1B). These faults and folds are indicative of the intense crustal shortening that occurred in these areas during the Cenozoic.

The crust of the Junggar Basin to the north is composed of Paleozoic island-arc complexes and overlaying Permian to Quaternary terrestrial deposits^33^. A Paleozoic ocean was located between the island-arc and the Yili Block to the south, and B-type subduction belts occurred along the southern and northern margins of the ocean^12^. Additionally, the crust of the Tarim Basin to the south consists of Mesozoic to Cenozoic terrestrial deposits, Paleozoic passive-margin sedimentary sequences and Precambrian basement^34^. Furthermore, a northward-directed late Paleozoic B-type subduction zone existed between the Tarim Basin and the Tianshan Mountians^9^,^10^,^11^,^12^,^14^.

The Cenozoic uplift of the Chinese part of the Tianshan Mountains is considered to be a distance-response to the collision of the Indian and Eurasian plates^17^,^23^,^29^,^35^,^36^,^37^,^38^. Various tectonic models of the uplift of these mountains have been proposed, including the subduction of basins on both sides of the mountains^39^, northward movements and the clockwise rotation of the Tarim Basin^22^,^40^, the northward indentation of the Pamir^15^,^32^, and mantle convection beneath the mountains under the compressional setting^41^. However, none of these models used a high-resolution data base of deep-seated structure and thus could not provide a satisfactory explanation for the origin of the unique geographical and geological features in these mountains.

## Receiver Function Images and the Deep Structure of the Profile between Urumuqi and Korla

**Figure 2:**
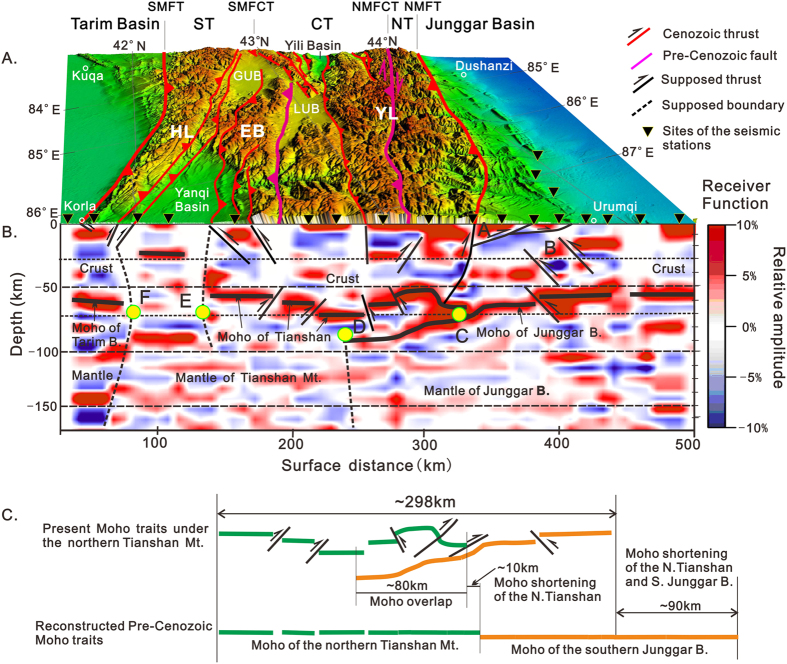
Receiver function image and its geological interpretation of Chinese Tianshan Mountains. (**A**) Geomorphologic features of Segment C in the Tianshan Mountains. (**B**) Receiver function image across the mountains along the highway from Urumuqi in the north to Korla in south and its geological interpretation; the red and blue colors show positive and negative stacking amplitudes, respectively. (**C**) Estimates of Cenozoic crustal shortening of northern part in the Tianshan Mountains. Abbreviations of the tectonic units and their boundaries near the surface as suggested by geologists: NT-North Tianshan, CT-Central Tianshan, ST-South Tianshan, NMFT-northern marginal fault of North Tianshan, NMFCT- northern marginal fault of Central Tianshan, SMFCT- southern marginal fault of Central Tianshan, SMFT- southern marginal fault of South Tianshan. Present-day ranges and basins: YL-Yilianhabierga Mt., EB-Erbin Mt., HL-Hola Mt., GYB-Great Yobulus Basin, LYB-Little Yobulus Basin. The primary DEM data that were used for the geomorphological features in (**A**) are in the SRTM GTOPO 30 format and were provided by NASA and downloaded from http://glcf.umiacs.umd.edu in 2010. The figure was generated by using ArcMap v10.1 (http://www.esrichina.com.cn/softwareproduct/ArcGIS/) and processed by CorelDRAW X7 (http://www.coreldraw.com/cn/product/graphic-design-software/?hptrack=cn2hr1&_ga=1.59748394.665041398.1450858064).

The receiver function method has been a main approach during the late twentieth century for understanding the deep structure of continental lithosphere^42^,^43^,^44^. The receiver function image of a profile across Segment C of the Tianshan Mountains along the highway from Urumuqi to Korla is one result of the broadband seismic probing project of eastern Xinjiang that was conducted out from May 2002 to November 2004^45^,^46^. Some new information regarding the deep structures of these mountains can be obtained from the images. Geological interpretations of the image are shown in Fig. 2B and are briefly discussed below.

Two obvious features are observed in the receiver function image (Fig. 2B). First, a strong P- to S-wave-conversion belt is present at depths of approximately 50–70 km below the surface, which is roughly concordant with the depth of the Moho as inferred from gravity anomalies^47^ and deep seismic reflection data^48^,^49^. Second, the crustal structure and tectonic features along the profile are heterogeneous and may be divided into three parts from north to south, i.e., the southern margin of the Junggar Basin, the northern part of the Tianshan Mountains, and the southern part of the Tianshan Mountains.

### Southern Margin of the Junggar Basin

The Moho under the southern Junggar Basin (north of point C in Fig. 2B) tilts to the south, transitioning from a depth of approximately 50 km in the southern Junggar Basin to a depth of nearly 70 km at the junction between the Junggar Basin and the Tianshan Mountains. The crust above the Moho is characterized by four structural layers, which are outlined in the receiver function images. The uppermost layer (point A in Fig. 2B) is a northward-thinning wedge with a north-verging imbricated-thrust structure. The other three layers are roughly parallel to the Moho. A layered structure below the Moho is also clear but becomes blurred to the south. Interestingly, south-verging thrusts that cut the Moho are present in the southern Junggar Basin (point B in Fig. 2B).

### Northern Part of the Tianshan Mountains

The Moho exhibits concave-upward deformation under the northern part of the Tianshan Mountains between the Junggar Basin to the north and the Yanqi Basin to the south (the region between points C and E in Fig. 2B), and an up-warp along the northern margin of the concavity. The Moho in the northern (right side of point D in Fig. 2B) and southern (left side of the point D in Fig. 2B) parts is located at approximately 50–60 km depth, whereas the Moho in the central part (point D in Fig. 2B) lies at a depth of approximately 75 km. Faults bound the northern and southern sides of the concavity, with thrust directions that are oriented toward the core of the mountains. This thrusting likely contributed to the Moho deformation and crustal shortening. The southward-dipping Moho of the Junggar Basin appears to underlie the Moho of the Tianshan Mountains in the region between points C and D (Fig. 2B), which contains the highest peak in Segment C of the Tianshan Mountains on the surface.

No clear layered structures can be seen under the Moho in the northern part of the Tianshan Mountians. However, a northward-dipping interface occurs at the mantle below the point D in Fig. 2B, possibly representing the southern boundary of the Junggar mantle.

### Southern Part of the Tianshan Mountains

The southern part of the Tianshan Mountains along the profile can be divided into two parts: the Yanqi Basin to the north and Hola Mt. to the south.

Under the Yanqi Basin (between points E and F in Fig. 2B), no Moho is connected to interfaces on either side, but two distinct transfer interfaces can be seen at depths of approximately 20 km and 80 km. The geological implications of these interfaces are not certain at present. A relatively weak converted wave surface is present between these two layers and tilted to the north, especially in the 40–50 km depth range. An even weaker converted wave surface occurs at approximately 60 km depth. Whether these two surfaces correspond to the modified Moho of the Tianshan Mountains (40–50 km) and Tarim Basin (60 km) remains to be investigated.

The receiver function image under the Hola Mt. region is characterized by an obvious layered structure. A strong conversion belt with a moderately northward dip is located at a depth of approximately 60 km. This feature is interpreted to be the Moho of the Tarim Basin.

## Estimates of Cenozoic Crustal Shortening

Previous investigations and estimates of the Cenozoic crustal shortening of the Tianshan Mountains and adjacent regions mainly examined in the piedmonts on the flanks of the range^23^,^24^,^25^,^26^,^31^. The relative lack of data from the interior of the mountains because of natural geomorphology constraints has hampered our knowledge of the crustal shortening in the mountains.

The available geological data reveal that the crust of the Tianshan Mountains and adjacent regions are a mosaic of Paleozoic island arcs and oceanic remains that were amalgamated during the Late Paleozoic^9^,^10^,^11^,^12^,^13^,^14^ and that peneplanation occurred at the end of the Jurassic^50^. According to the features in the studied image, we suppose that the present Moho likely formed following the peneplanation at the end of the Jurassic and that its dislocations and overlap are records of intracontinental orogenesis during the Cenozoic. Thus, using the Moho as a key marker bed provides a new approach to study and estimate the Cenozoic crustal shortening in this region.

As shown in Fig. 2C, we obtained a Cenozoic crustal shortening estimate of ~90 km for the northern part of the Tianshan Mountains and its northern adjacent region according to the above suppositions and by using the balanced section technique from structural geology^51^. In detail, approximately 80 km of the shortening originated from the overlap of the Moho between the northern Tianshan Mountains and the Junggar Basin, and the remaining 10 km originated from the dislocation and bending deformation of the Moho under the northern Tianshan Mountains. However, the above estimate is probably the minimum shortening of the crust in the northern part of the Tianshan Mountains because this feature originated from the deformation of the Moho in the north segment of the 2-D seismic profile and because we do not know the detail of the deformation of the Moho.

## Discussion and Conclusions

The northern part of the above receiver function image is reliable and authentic because of the high quality of the collected seismic data, and the rationality of data-processing. However, some uncertainty is present for its southern part because of natural disturbances from the thicker Cenozoic sediments in the Yanqi Basin. However, the difference in the deep structures between the northern and southern parts of the mountains should be an objective fact based on the available seismic data. For example, Shao *et al.*^52^ reported that thicker crust and more complex deep-seated structures are present in the northern part of the same profile, and no trace of Paleozoic subduction or plate collision is present in the deep structures alone the profile. However, these authors did not separate Cenozoic structures from Paleozoic structures, and reached the conclusion that neither subduction nor collision between plates occurred in the region during the Phanerozoic. The cut-off and lifting but absence of overlap of the Moho below the Yanqi Basin in the southern part of the profile can be observed in the image that was published by Mi *et al.*^53^, which indicated thinner crust in the basin compared to that on either side. However, we did not deduce any information from the image regarding the subduction of the Tarim lithosphere under the basin during the Cenozoic as supposed by Mi *et al.*^53^.

Another highway through the Tianshan Mountains is present approximately 200 km westwards from the above profile. Some seismic investigations into the deep structures of the mountains have been performed along this highway. The deep structural profiles that were obtained by these investigations^54^,^55^,^56^,^57^,^58^ showed that the crust in the northern Tianshan Mountains is thicker and characterized by more complex structures; below the crust, is a cut-off in the Moho and a southward-dipping layer of high velocity. A high velocity archy-like layer occurs in the lithosphere below the southern parts of the mountains, and a nearly vertical velocity structure exists in the junction between the Tarim Basin and the Tianshan Mountains.

The deep-source earthquakes in this region reveal the occurrence of structural deformation near the Moho under the northern part of Segment C in the Tianshan Mountains^39^,^59^. The surface structural deformation, including thrusts along the margins of the intermontane basins within the Tianshan Mountains, folds and thrusts in Mesozoic and Cenozoic strata in the northern piedmont of the mountains, and numerous earthquakes with hypocenters at depths of 20 km or less, implies the occurrence of intense crustal tectonic stacking and shortening in the region. Additionally, intracrustal detachment at approximately 20–30 km depth and imbricated-thrust structures at 0–12 km depth under the southern Junggar Basin have been revealed by two deep seismic reflection profiles^48^,^49^. In contrast, no folds or thrusts that resemble those along the southern margin of the Junggar Basin occur in the southern piedmont region of Segment C in the mountains.

In summary, available deep-seated structural data from Segment C in the Tianshan Mountains show a significant difference between northern and southern parts of the mountains, namely, a deep-seated structure, which corresponds to the region’s geomophological features. This deep-seated structure beneath the high linear ranges of the northern part is characterized by more complex intracrustal deformation, thicker crust, concave-upward geometry in the Moho, and Moho overlap between the northern Tianshan Mountains and the Junggar Basin. Below the alternate ranges and basins of the southern part of the mountains are simpler intracrustal structures, a nearly flat, local cut-off but no overlap of the Moho, and very limited indentation of the Tarim lithosphere.

The different responses of the southern and northern parts of the mountains to a compressional setting from the collision of the Indian and Eurasian plates may be related to their crustal tectonics and the geometric relationship of the regional compressional stress with respect to the boundaries between the basins and mountains. The crust in the northern part is composed of linear arc complexes to the south and accretionary complexes to the north. The strikes of these complexes and the boundary between the Junggar Basin and the Tianshan Mountains are nearly perpendicular to the direction of the Cenozoic regional compressional stress^60^,^61^ (Fig. 1B). In contrast, the crust in the southern part of the Tianshan Mountains consists of a mosaic of arc and oceanic complexes, and structural styles such as boudinage developed more readily under plane compressional stress in the south-north direction. The boundary between the Tarim Basin and the Tianshan Mountains forms an arc-like line that protrudes northwards, and most parts of the boundary are oblique to the direction of the Cenozoic regional compressional stress (Fig. 1B). Simultaneously, the northward indentation of the Pamir range induced the clockwise rotation of the Tarim Basin. These factors contributed to the development of different local stress fields in the various segments of the boundary.

**Figure 3:**
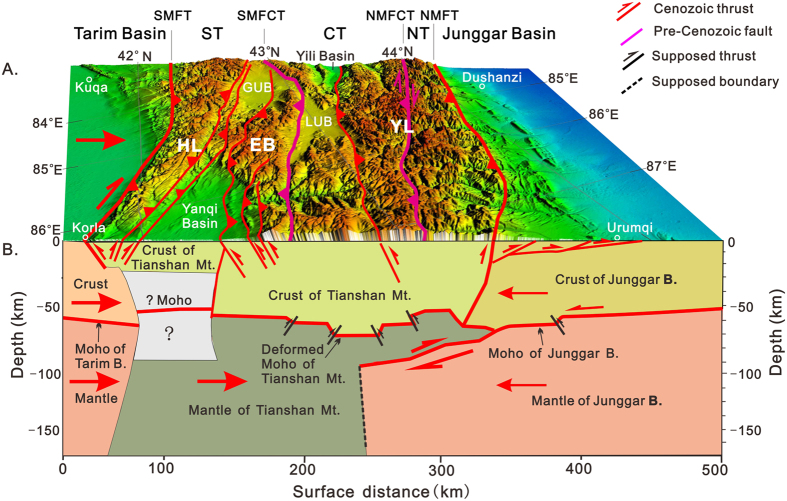
Cartoon map of Segment C in the Tianshan Mountains. (**A**) Geomorphologic features of Segment C in the Tianshan Mountains. (**B**) Deep structures and the driving mechanism for the uplift of the mountains during the Cenozoic. The abbreviations are the same as those in Fig. 2. The primary DEM data that were used for the geomorphological features in (**A**) are in the SRTM GTOPO 30 format and were provided by NASA and downloaded from http://glcf.umiacs.umd.edu in 2010. The figure was generated by using ArcMap v10.1 (http://www.esrichina.com.cn/softwareproduct/ArcGIS/) and processed by CorelDRAW X7 (http://www.coreldraw.com/cn/product/graphic-design-software/?hptrack=cn2hr1&_ga=1.59748394.665041398.1450858064).

Based on the above discussion, we propose a new tectonic model for the Cenozoic intracontinental orogenesis of Segment C in the Tianshan Mountains, as shown in Fig. 3. Nearly north-south-oriented stress that originated from the collision of the Indian and Eurasian plates moved the Tarim block northward, pushing the Tianshan lithosphere northward as well. The limited indentation of the Tarim lithosphere into the southern margin of the Tianshan Mountains produced unique geomorphology. The lithosphere of the Junggar Basin uncoupled along the Moho, and its mantle was subducted beneath the Tianshan Moho. This process led to shortening deformation in the Tianshan Moho, the structural stacking and thickening of the overlying crust, and the uplift of high, linear mountain ranges in the northern part of the Tianshan Mountains. Simultaneously, the Tianshan mantle was inserted northwards along the Moho into the lithosphere of the Junggar Basin, forming a crocodile mouth-like structural framework at the junction of the Tianshan Mountains and the Junggar Basin. This model, in terms of its size, deep-seated structures, surface geology and geomorphology, especially when considering only the subduction of the mantle part of the lithosphere, is clearly different from the B-type subduction at active continental margins and A-type subduction at collisional belts between two continents. Thus, we suggest that the above model be termed mantle subduction and abbreviated M-type subduction.

The overlap of the Moho beneath the northern part of Segment C of the Tianshan Mountains is not interpreted as a re-activated Paleozoic subduction zone mainly because of the difference in the dipping direction between fossil subduction and the present overlapping Moho and because of the relationship of the deep structures with near-surface ancient tectonics. The available regional geological data show four Paleozoic subduction zones with various subducted polarities in the Tianshan Mountains, and a subduction zone with northward polarity was located south of the Junggar island arc, which has acted as the basement of the Junggar Basin since the Permian^11^,^62^. Traces of all the subduction zones are not always observed in all the available deep seismic profiles, indicating that these Paleozoic subduction tectonics were only recorded in rocks in the upper part of the crust.

Some geologists have suggested a crustal material cycle model in which erosion drove the uplift of the Tianshan Mountains^2^,^6^. For this driving mechanism, the geomorphological differences would have to have existed prior to erosion. Therefore, thicker crust and high mountains would need to be already present in the erosion regions, and relatively thinner crust and lower-elevation topography would need to be present in the sedimentary region. The development of these geomorphic differences is not addressed in their models. Uplift that was driven by mantle subduction possibly preceded the crustal material cycle.

High linear mountains that are associated with fold-and-thrust deformation in piedmont regions and are similar to those in Segment C of the Tianshan Mountains are also present in Segment B and possibly in Segment A of the mountains (Fig. 1B). Up to now, images of deep structures have only been reported from Segment B. Earlier investigations of seismic tomography did not uncover any information regarding mantle subduction^63^,^64^,^65^,^66^. However, the overlap of the Moho was revealed by a recent N-S-directed explosive-source deep seismic-reflection profile in the southern piedmont region of Segment B in NW Tarim Basin^67^, which indicated small-scale (less than 20 km) mantle subduction. In addition, similar Moho overlaps have been reported from the western Qinling and Longmenshan ranges on the northeastern margin of the Qingzang Plateau^68^,^69^,^70^. Furthermore, decoupling along the Moho has been reported beneath some mountain ranges elsewhere on Earth^71^, which implies that mantle subduction likely occurred below those mountains. Simulations of the origin of mountains also reveals similar mantle subduction^72^. This information and data suggest that mantle subduction should be a primary driving mechanism for the uplift of high, linear intracontinental mountains under compressional stresses that are nearly perpendicular to the mountains’ strike.

## Method

The field collection of a seismic dataset was conducted between the May 2002 and November 2004, producing a receiver function image of the profile across the Chinese Tianshan Mountains along the highway from Urumuqi to Korla, as part of a broadband teleseismic investigation of eastern Xinjiang from Altay southwards through East Junggar, the Junggar Basin and the Tianshan Mountains to the northern Tarim Basin,. Refteck and Minittan’s seismographs which used band ranges of 60–5 s, were used to obtain the dataset. The sites of the seismic stations across Segment C of the Tianshan Mountains were spaced at an interval of 10–15 km, as shown in Fig. 1B. The data processing and formation of the images were conducted by using the same methods as Kind *et al.*^73^, and the details have been discussed by Li *et al.*^45^.

## Additional Information

**How to cite this article**: Li, J. *et al.* Mantle Subduction and Uplift of Intracontinental Mountains: A Case Study from the Chinese Tianshan Mountains within Eurasia. *Sci. Rep.* **6**, 28831; doi: 10.1038/srep28831 (2016).
